# Complements C3 and C5 Individually and in Combination Increase Early Wound Strength in a Rat Model of Experimental Wound Healing

**DOI:** 10.1155/2013/243853

**Published:** 2013-05-23

**Authors:** Hani Sinno, Meenakshi Malhotra, Justyn Lutfy, Barbara Jardin, Sebastian Winocour, Fadi Brimo, Lorne Beckman, Kevin Watters, Anie Philip, Bruce Williams, Satya Prakash

**Affiliations:** ^1^Division of Plastic and Reconstructive Surgery, Department of Surgery, McGill University, Montreal, QC, Canada H3G 1A4; ^2^Biomedical Technology and CellTherapy Research Laboratory, Department of Biomedical Engineering, Faculty of Medicine, McGill University, 3775 Rue University, Room 311, Lyman Duff Medical Building, Montreal, QC, Canada H3A 2B4; ^3^Department of Pathology, McGill University, Montreal, QC, Canada H3G 1A4; ^4^Orthopedic Research Laboratory, McGill University, Montreal, QC, Canada H3A 1A1

## Abstract

*Background*. Complements C3 and C5 have independently been shown to augment and increase wound healing and strength. Our goal was to investigate the combinatorial effect of complements C3 and C5 on wound healing. *Methods*. Each rat served as its own control where topical collagen was applied to one incision and 100 nM of C3 and C5 in collagen vehicle was applied to the other incision (*n* = 6). To compare between systemic effects, a sham group of rats (*n* = 6) was treated with collagen alone on one wound and saline on the other. At day 3, the tissue was examined for maximal breaking strength (MBS) and sectioned for histological examination. *Results*. There was a statistically significant 88% increase in MBS with the topical application of C3C5 when compared to sham wounds (*n* < 0.05). This was correlated with increased fibroblast and collagen deposition in the treated wounds. Furthermore, there appeared to be an additive hemostatic effect with the C3C5 combination. *Conclusions*. The combination of complements C3 and C5 as a topical application drug to skin wounds significantly increased wound healing maximum breaking strength as early as 3 days.

## 1. Introduction

Wound healing can be problematic in several clinical settings. The current available surgical and medical options are not always ideal for all patients. The development of a novel therapeutic agent that may help augment the healing process is urgent. An understanding of the intricate cascade of events and cellular interactions is essential in the development of such therapeutic agents.

The complement cascade involves the interaction and cleavage of eleven proteins to form complexes responsible for hemostasis, chemotaxis, and bacterial lysis [1]. Complements C3 and C5 have independently been shown to play a role in wound healing, augmenting wound strength, and increasing cellular infiltration and collagen deposition [2, 3]. Furthermore, C5 has been shown to accelerate healing by at least four days in the first week of wounding [3]. The synergistic effect of combining C3 and C5 to augment wound healing is not yet known.

We aim to decipher the potential synergistic effects that C3 and C5 may have on wound healing strength. Our objective is to combine C3 and C5 as a topical therapeutic agent to assess changes in wound strength and cellular infiltration. The potential synergistic effect on wound healing can be potentially a great advancement in the understanding of the complex processes of wound healing and translation to a novel therapeutic agent for the use and benefit for patients.

## 2. Materials and Methods

### 2.1. *In Vivo* Experimental Study

All procedures performed were in accordance with the guidelines of the McGill University Committee on Use and Care of Animals and have been approved by the McGill Animal Ethics Committee and Veterinary Care Services. Adult male Sprague-Dawley rats, 300 to 350 g (Charles River; Saint Constant, QC, Canada), were housed one week prior to surgery for acclimatization in clean separate cages and fed ad libitum water and standard rat chow. This study included two groups of experiments.

#### 2.1.1. Group I: Sham Rats

This group (*n* = 6) was used as a control group. Type I collagen was purchased from PureCol (Inamed BioMaterials; Fremont, CA, USA) at concentrations of 6 mg/mL. The pH was adjusted to 4–4.5 by addition of 0.1 M NaOH. Collagen was chosen as a vehicle medium because of its relative inert nature and viscous properties that allows for a theoretical slow-release system [4]. The experimental wounds in the sham rats received collagen and the control side received normal saline (0.9% NaCl, 300 mOsm/L). 1 cc volume of solution was added to each incision.

#### 2.1.2. Group II: C3C5 Rats

In the second group (*n* = 6), the synergistic effect of a combination of C3 and C5 in collagen solution was tested. C3 and C5 were purchased from VWR International (Montreal, Canada) as a stock solution of 250 *μ*g/mL and were added to the collagen solution at concentrations of 100 nM each and were topically applied to the experimental wound and collagen alone on the control wound. 1 cc volume of solution was added to each incision.

#### 2.1.3. Rational for Group Design

Group I rats were used as controls to compare with Group II experimental rats. Each group had two separate incisions. Each incision was treated with a different formulation. In Group I rats, one incision was treated with saline and the other was treated with collagen. Group I rats were not treated with any complement formulation. In Group II rats, one incision was treated with the C3C5 Collagen formulation and the other incision in only collagen formulation (no C3C5 was present). Any changes to wound strength, scar formation, histology, and protein content in Group II rats would be likely secondary to the presence of the C3 and C5 complements as Group I did not have any C3 or C5 treatment. In Group II, If only the C3C5 incisions showed increases in wound strength and not the collagen-treated incisions, then C3C5 does not have a systemic effect and only a local effect. The synergistic wound healing effects of C3 and C5 (Group II) were compared to the individual treatments of C3 (Sinno et al. [2]) and C5 (Sinno et al. [3]). All incision treatments were blinded and randomized to help eliminate any bias.

### 2.2. Surgical Protocol

Isoflurane gas (4-5% for induction, 1-2% for maintenance) and subcutaneous injection of Carbofen (5 mg/kg) were used to anesthetize the rats. The dorsum was shaved with an electric hair clipper and disinfected with 70% alcohol. Two full-thickness 6 cm linear skin incisions were made in the median plane (2 cm on either side of the midline), beginning 1 cm below the inferior edge of the scapula using sterile no. 10 surgical scalpels [5]. One wound received a single topical application of control solution and the other wound received the same volume of the experimental solution. This allowed each rat to serve as its own control. The incisions were reapproximated with five equidistant surgical clips, and the rats were monitored under heat lamp for an hour postoperatively. Animals were sacrificed on Day 3 using inhaled carbon dioxide gas followed by cervical dislocation. The central 3 cm of the wounds were harvested for tensometry measurements and the outer 3 cm strips were prepared for histological analysis.

### 2.3. Blood Analysis

To determine any measurable systemic effects on complement serum levels and inflammatory markers, blood tests were conducted during the recovery period at the day of surgery and on the day of sacrifice. A complete blood count and differential were measured looking at the cell count at day 3 as compared to that of the wounding day. Inflammatory markers in the serum such as CRP, C3, and C4 were examined to determine any measurable systemic changes at the different time points. Blood samples were retrieved from the right saphenous vein during the recovery period on the day of wounding and just before sacrifice.

### 2.4. Tensometry

The maximum wound breaking strength (MWBS) was calculated from three 10 mm strips from each wound (*n* = 18) with a tensometer (Tensometer 10; Monsanto Co., St. Louis, MO, USA). The 10 mm strips were precisely cut using a preformed instrument with two microtome blades separated by a 10 mm thick steel beam. The skin strips were placed vertically between the clamps of the tensometer with the wound at the center (2 cm from each jaw). A force was applied with a constant speed of 10 mm/s until rupture. The forces were plotted on computer software, and the MWBS was measured as the greatest force before rupture of the wounds. Tensile strength is the forces per unit of cross-sectional area. Since the cross-sectional area was made constant in all skin strips (10 mm wide, 40 mm jaw space, and similar adjacent skin width), the MWBS is directly proportional to tensile strength and is used interchangeably in this discussion.

### 2.5. Histopathology

At the time of sacrifice, the edges of the wounds and any open wounds not utilized for tensometry were excised for histological preparation. Three 5 mm perpendicular sections were placed in a tissue cassette between biopsy sponges. The specimens were then fixed in 10% Formalin, processed, and embedded in paraffin. The skin surface was identified and the specimens were cut at 5 *μ*m intervals perpendicular to the long axis of the wound surface using an Olympus microtome. Hematoxylin and eosin staining was utilized to visualize the gross microscopic cellular architecture. A blinded pathologist examined the slides for assessment of healing and cellular infiltration. A grading scheme was utilized to quantify differences between the specimens: Grade I, few fibroblast infiltrates; Grade II, moderate fibroblast infiltration; and Grade III, maximal fibroblast infiltration. The same grading scheme was used to assess the extent of inflammation and fibrosis in the wounds.

### 2.6. Statistical Analysis

A paired Student's *t*-test was utilized to compare means of experimental data between the same rats. The unpaired *t*-test was utilized to compare mean values of experimental data of different rats. A *P* value of <0.05 was considered statistically significant. The data is expressed as means ± SE.

## 3. Results

### 3.1. Tensometry Analysis

The blinded subjective assessment of the cosmetic appearance of the scars (raised borders, color, width, and general appearance) demonstrated no differences between the collagen-treated wounds as compared to the C3C5-treated wounds at all time points. Furthermore, there were no differences in the cosmetic appearance when Group I wounds were compared to Group II wounds at Day 3.

**Figure 1:**
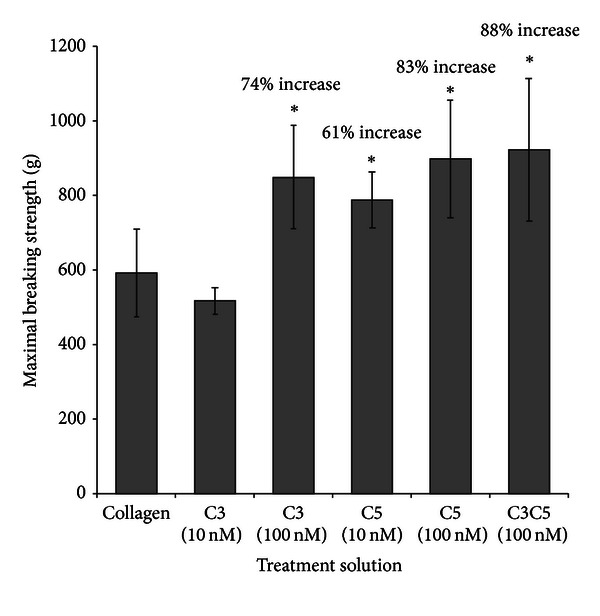
Maximal wound breaking strength of treated wounds as compared to control wounds at Day 3. In the horizontal axis, “collagen” represents the tensometry of the collagen treated wounds in the sham rats. The C3 (100 nM) [2], C5 (10 nM) [3], and C5 (100 nM) represent the tensometry of the complement (concentration) in collagen solution on experimental wounds. The C3C5 (100 nM) represents the combination of complements C3 and C5 in collagen formulation at concentrations of 100 nM on experimental wounds. Treatment of wounds with C3 (100 nM), and C5 (10 nM), C5 (100 nM) has been shown previously to increase MWBS as compared to sham wounds. The increase of MWBS attained with the application of C3C5 in combination does not seem to significantly differ than with the administration of C3 and C5 alone. A significant increase of 88% in maximal wound breaking strength can be seen with topical formulation of C3C5 at concentrations of 100 nM at Day 3. *P < 0.05.

Mechanical analysis was then performed on the wounded skin. Values for maximal wound breaking strength (MWBS) are presented in Table 1. Values are presented in grams and are proportionally representative of wound tensile strength. *Group II*. C3C5 Rats: the synergistic effect of C3 and C5 was measured on MWBS in rat wounds. There was no difference in MWBS between the C3C5-treated wounds (923 ± 191 g) as compared to the contralateral collagen-treated wounds (787 ± 93 g) at Day 3 in the same rat. However, a significant increase in the MWBS of C3C5-treated wounds was measured when compared to the collagen treated wounds in sham rats (*P* < 0.05) Figure 1. This translated into an 88% increase in wound tensile strength. Furthermore, the MWBS of the collagen-treated wounds in Group II rats (787 ± 93 g) was significantly higher than that in the collagen-treated wounds in Group I rats (490 ± 57 g), translating to a 61% increase in tensile strength (*P* < 0.05).

### 3.2. Comparison of C3, C5, and C3C5

C3 and C5 have previously shown to increase MWBS as compared to sham wounds [2, 3]. Figure 1 illustrates that treatment of wounds with C3 100 nM [2], C5 10 and 100 nM [3], and C3C5 100 nM significantly increases MWBS as compared to the collagen-treated wounds in the sham rats at Day 3. No statistical difference is seen when a comparison between Day 3 MWBS of C3 100 nM, C5 10 nM, C5 100 nM, and C3C5 100 nM treated wounds was performed.

To determine any systemic hematologic or inflammatory effects from the topical application of complement, CBC, CRP, C3, and C4 blood levels were analyzed after the surgery and the day of sacrifice. Blood counts of neutrophils, lymphocytes, monocytes, and eosinophils were not different when compared between Day 0 and Day 3. Platelet count increased from day 0 (626 ± 193) to day 3 (1137 ± 80) in the C3C5-treated rats (*P* < 0.05) as was seen in the sham group. No differences were found in all the inflammatory markers (CRP, C3, and C4) and CBC when C3C5- (Group II) treated rats were compared with sham rats (Group I) except for haemoglobin levels on day 3. The C3C5-treated rats showed a higher haemoglobin content than sham rats at Day 3 after wounding (160 ± 3.3 versus 146 ± 2.2, resp., *P* < 0.05).

**Figure 2:**
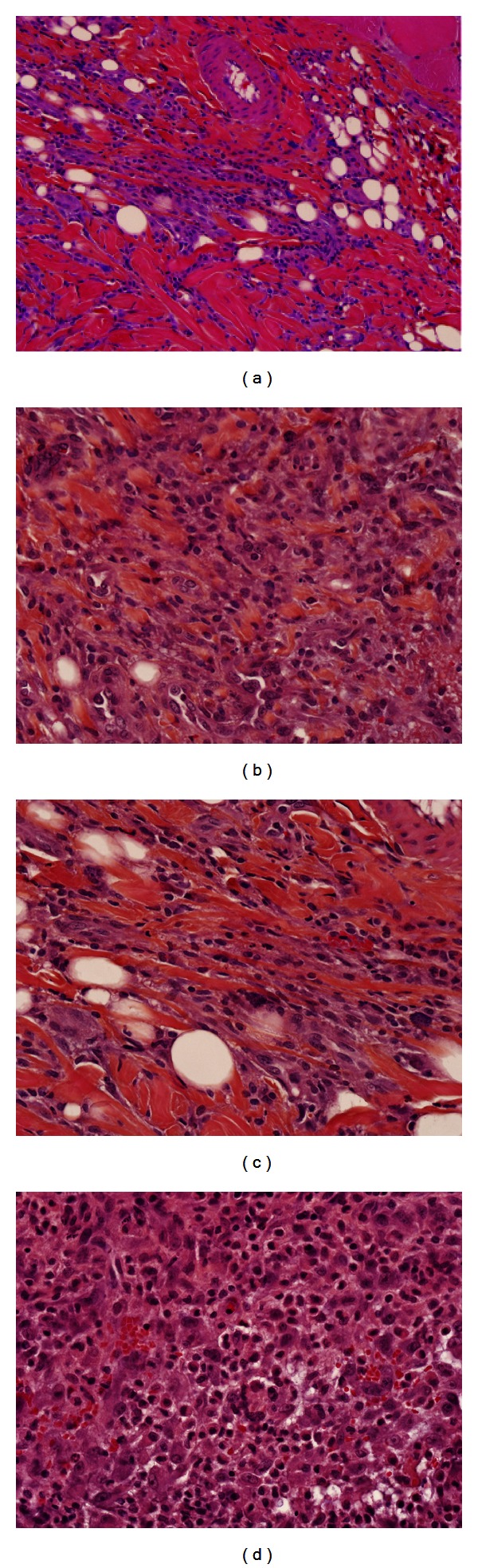
Histological imaging of control and experimental wounds stained with H&E. Blue represents nuclear staining. (a) 20x magnification of a representative control wound at Day 3; (b) 20x magnification of a representative C3C5-treated wound at Day 3; (c) 40x magnification of a representative control wound at Day 3; (d) 40x magnification of a representative C3C5-treated wound at Day 3. There is an increased cellular infiltration in the experimental wounds ((b) and (d)) as compared to control wounds ((a) and (c)) as represented by an increased inflammatory cell nuclear staining. It is evident that an increased extent of inflammation in the C3C5-treated wound beds exists as seen in the lower-power images. There is also an obvious increased collagen deposition and organization in the experimental wounds as seen in higher power images.

The histological analysis was utilized to determine any cellular effects that the C3C5 treatments may have had on wound healing. There was a trend toward a greater number of cellular infiltration and fibroblast deposition and collagen content in the C3C5-treated wounds as compared to the control rats (Figure 2).

## 4. Discussion

We have previously shown the role of complements C3 and C5 on wound healing [2, 3]. The topical application of either complement on wounds was associated with an increase in wound strength, fibroblasts infiltration, fibronectin, and collagen deposition [2, 3]. In the current study, we attempted to decipher a synergistic role of the application of both C3 and C5 on wounds.

The combination of C3 and C5 at doses of 100 nM showed significant increases in wound strength of up to 88% as compared to control rats. When C3C5-treated wounds were compared to C3-treated wounds and C5-treated wounds, there seemed to be no statistical differences in MWBS. The combination of C3 and C5 did not show a significant additive effect although there was a trend toward such a difference. The combination of C3 and C5 to wound treatment did not show any significant hematologic or inflammatory changes as compared to sham rats except for an increase in haemoglobin level at Day 3. This increase in haemoglobin is a desirable postoperative feature. One explanation can be related to the haemostatic effect that the complement system has. It seems that combining C3 and C5 may lead to an increase in haemostasis at the time of wounding leading to reduction in intraoperative blood loss, which was visually appreciated but difficult to quantify. Clinically, this may translate to decreases in operative related bleeding and subsequent transfusions.

The application of C3C5 on one wound appeared to increase the wound strength of all wounds within the same rat. There appears to be a systemic effect of the topical application of C3 and C5 to increase wound strength, but this effect does not seem to be additive. This is a desirable feature in the development of a wound healing drug, as all wounds in the body seem to heal faster and stronger. As inflammatory levels including serum C3 levels do not seem to be different between the experimental groups, the cleaved products of C3 and C5 may be diffusing towards the adjacent wounds or even entering the blood stream resulting in the observed systemic wound healing effects. This whole-body healing effect of C3 and C5 seems to be primarily during the first phases of wound healing. It is known that C3 and C5 are spontaneously activated and help form a haemostatic plug in the first seconds of wound formation. Soon after, C3 and C5 become cleaved into smaller proteins that mobilize to form the membrane attach complex and are directly responsible for the opsonisation and death of foreign microorganisms. In addition, early in the inflammatory and proliferative phases of wound healing, C3 and C5 are active in inflammatory cellular chemotaxis. Their primary role in increasing vascular permeability may further accelerate healing by promoting cellular infiltration.

## 5. Conclusion

Our novel approach to the treatment of wounds with the topical application of complements C3 and C5 is based on the original idea that complements have a normal physiologic reaction in wound healing responsible for haemostasis, microorganism lysis, and inflammatory cell recruitment. We found a trend towards an additive effect on wound strength with the C3C5 combination topical formulation. Furthermore, there appeared to be an additive haemostatic effect leading to a significant decrease in the reduction of postoperative hemoglobin. The increase in wound healing and additive haemostatic effects further support the use of the combination of complements C3 and C5 as a therapeutic agent for incisional wounds.

## Figures and Tables

**Table 1:** Effect of the topical application of collagen formulation and the combination of complements C3 and C5.

		Side	Maximal wound breaking strength (g)
Group I	Sham	Collagen	490 ± 57
		Saline	478 ± 86

Group II	C3C5	Collagen	787 ± 93*
		C3C5 in collagen	923 ± 191*

*P < 0.05, calculated using the unpaired *t*-test comparing the MWBS means of experimental wounds (Group II) to those of the Sham wounds (Group I).
